# A Robust Design for Aperture-Level Simultaneous Transmit and Receive with Digital Phased Array

**DOI:** 10.3390/s22010109

**Published:** 2021-12-24

**Authors:** Mingcong Xie, Xizhang Wei, Yanqun Tang, Dujuan Hu

**Affiliations:** School of Electronics and Communication Engineering, Sun Yat-Sen University, Xinhu Street, Guangming District, Shenzhen 518107, China; xiemc3@mail2.sysu.edu.cn (M.X.); tangyq8@mail.sysu.edu.cn (Y.T.); hudj@mail2.sysu.edu.cn (D.H.)

**Keywords:** aperture-level simultaneous transmit and receive (ALSTAR), adaptive beamforming, adaptive random group quantum brainstorming (ARGQBSO), digital phased array, robust design

## Abstract

Aperture-level simultaneous transmit and receive (ALSTAR) attempts to utilize adaptive digital transmit and receive beamforming and digital self-interference cancellation methods to establish isolation between the transmit and receive apertures of the single-phase array. However, the existing methods only discuss the isolation of ALSTAR and ignore the radiation efficiency of the transmitter and the sensitivity of the receiver. The ALSTAR array design lacks perfect theoretical support and simplified engineering implementation. This paper proposes an adaptive random group quantum brainstorming optimization (ARGQBSO) algorithm to simplify the array design and improve the overall performance. ARGQBSO is derived from BSO and has been ameliorated in four aspects of the ALSTAR array, including random grouping, initial value presets, dynamic probability functions, and quantum computing. The transmit and receive beamforming carried out by ARGQBSO is robust to all elevation angles, which reduces complexity and is conducive to engineering applications. The simulated results indicate that the ARGQBSO algorithm has an excellent performance, and achieves 166.8 dB of peak EII, 47.1 dBW of peak EIRP, and −94.6 dBm of peak EIS with 1000 W of transmit power in the scenario of an 8-element array.

## 1. Introduction

The discussions about 5G, mm-wave, and MIMO technologies have never slowed their pace, which implies that existing wireless communication system throughput is still far from meeting the actual demands. Fortunately, simultaneous transmit and receive (STAR) technology (i.e., transmitting and receiving at the same time in the same frequency band) has been considered a reliable way to overcome this trouble, which is the performance of potentially doubling the capacity or spectral efficiency compared to traditional TDD and FDD [1]. In fact, STAR was originally used in frequency modulated continuous wave radar to achieve stealth by continuously illuminating the target with a low-power waveform. With the active exploration of STAR by researchers, its superiority and competitiveness are recognized by scientists in other fields. It has been considered in electronic warfare systems as a means to continually detect weak signals in strong interference [2]. Furthermore, STAR is also widely used in multifunctional vehicle systems and military and civilian airports to achieve communication, sensing, and surveillance [3] simultaneously.

However, the implementation of STAR technology must depend on its sufficient isolation between the transmitter and the receiver. Limited by the compact space between the transmitter and the receiver, self-interference signals will inevitably be generated when the transmitter is running, which causes the receiver to be blocked or saturated. Thus, how to cancel the self-interference is urgent for the STAR system. Until now, many methods are best classified by the domain in which they operate, such as the propagation domain, analog domain, and digital domain [4]. Yet the methods in the propagation domain normally achieved only 30~40 dB of isolation in a narrow bandwidth [5] by antenna separation, antenna orientation, and placing absorptive shielding. Due to the use of active attenuators, phase shifters, and delayers, the analog-domain cancellation methods introduced inestimable non-linear distortion and they are often power-intensive, expensive, and physically large [6]. Digital-domain methods include digital self-interference cancellation, transmitting and receiving beamforming, channel estimation, and fingerprint signals. These approaches will be flexible, efficient, and reliable if the signal of interest is effectively received within the dynamic range of ADCs and DACs. Ahmed et al. proposed an all-digital nonlinear estimation and self-interference cancellation technology to improve the isolation above 20 dB in a single-receiving and single-transmitting full-duplex system [7]. Qiu et al. verified that adaptive beamforming provides a high isolation between the transmitter and the receiver [8]. They optimized the beamformers using the linear constrained minimum variance algorithm to provide at least 110 dB of isolation, without affecting target detection. Liang et al. proposed a method to realize adaptive transmit beamforming together with digital SIC [9], which provides positive inspiration for the advancement of digital cancellation.

Aperture-level simultaneous transmit and receive (ALSTAR) based on a digital phased array was proposed by the MIT Lincoln Laboratory in 2016 [10]. ALSTAR integrates digital cancellation and transmit and receive beamforming technologies to achieve extremely high isolation, termed effective isotropic isolation (EII). Since the theory is immature, a lot of work is still needed to extend the method to practice. For instance, effective isotropic radiated power (EIRP) and effective isotropic sensitivity (EIS) of the system should be taken into account to ensure a high emission efficiency and receiver performance. The increase of target parameters complicates the design of the STAR array. In this case, the designers wish to make a trade between EII, EIRP, and EIS to meet the requirements of different scenarios. The authors in [11] used an alternate optimization (AO) to design the EII of the ALSTAR array and then loaded a weight diagonally in the noise covariance matrix to reveal the relationship between EII and the gain. Yet this approach only analyzes the connection between them, and does not study how to trade EII, EIRP, and EIS. Furthermore, the AO algorithm needs to construct identities of two objective functions for alternate iteration updates in two directions, and perform a beamformer optimization at every elevation angle, which increases the computational complexity and hardware cost. This may limit the further promotion and application of ALSTAR.

This paper is dedicated to the improvement of the overall performance of the ALSTAR array and the reduction of complexity. We trade EII, EIRP, and EIS by proposing the weight $w_{f}$ to enhance the overall performance and optimize a set of transmitting and receiving beamformers independent of the elevation angle in order to reduce complexity. We found that the swarm intelligence optimization methods have the potential to achieve these goals. They have been successful in the fields such as beamforming-based pattern synthesis [12], array optimization [13,14], DC brushless motor efficiency problems [15], Loney’s solenoid problem [16], and stock index forecasting [17]. Extensive literature reveals that compared to traditional particle swarm optimization (PSO), genetic algorithm (GA), and differential evolution (DE), the brainstorm optimization (BSO) algorithm [18] has the characteristics of fast convergence, excellent robustness, and a strong global optimization ability in solving non-convex, multi-objective, and multi-modal optimization problems. This algorithm is quite suitable for the design of the ALSTAR array, but there is still space for improvement in the BSO algorithm [19].

In the classic BSO algorithm, each individual in the population may become a potential solution, which corresponds to a new idea in the process of human brainstorming. The process can be summarized in three steps. First of all, a blocking method similar to k-means is used to group individuals in the population, and the individual with the best fitness value will be the center of each block. Next, a new individual will be obtained by interacting information between individuals in one or more blocks. At last, the logarithmic sigmoid function with Gaussian random is used as the step size to update the individual. Correspondingly, the drawbacks of the classic BSO algorithm include k-means grouping with complex calculations, poor individual creation methods, and suboptimal update mechanisms.

In view of these shortcomings of BSO, we propose the adaptive random grouping quantum BSO (ARGQBSO) algorithm and apply it to design the ALSTAR array. The improvements of ARGQBSO are made by presetting initial value, random grouping, dynamic probability function, and quantum computing. Experimental results show that the preset initial value shortens the search range and speeds up the convergence of the algorithm. Random grouping reduces the complexity of the algorithm. The dynamic probability function and quantum update improve the accuracy of the algorithm. In terms of algorithm architecture, our innovations are mainly manifested in two aspects.

(1) According to the demands of the ALSTAR array, the weight $w_{f}$ is put forward to trade EII, EIRP, and EIS. Its significance is to enable the performance of the ALSTAR array to meet the needs of EII, EIRP, and EIS in various scenarios.
(2) The proposed ARGQBSO algorithm aims to achieve digital self-interference cancellation and adaptive beamforming. By proposing preset initial values and improving random grouping, dynamic probability functions, and quantum updates, the algorithm is a better balance in solving accuracy, solution time, and robustness.
(3) The beamformer optimized by ARGQBSO is independent of an angle and can be applied to any scanning angle. Its advantage is that the resources of the digital chip are greatly saved.

The remainder of this paper is organized as follows. In Section 2, the signal model and optimization model of the ALSTAR array are given. Section 3 introduces the origin of the ARGQBSO algorithm and detailed improvement measures. Section 4 discusses and analyzes the results of the six algorithms in the ALSTAR application, and verifies the competitiveness of the proposed algorithm. Finally, the conclusion and future work are provided in Section 5.

## 2. System Model

### 2.1. Signal Model

**Figure 1:**
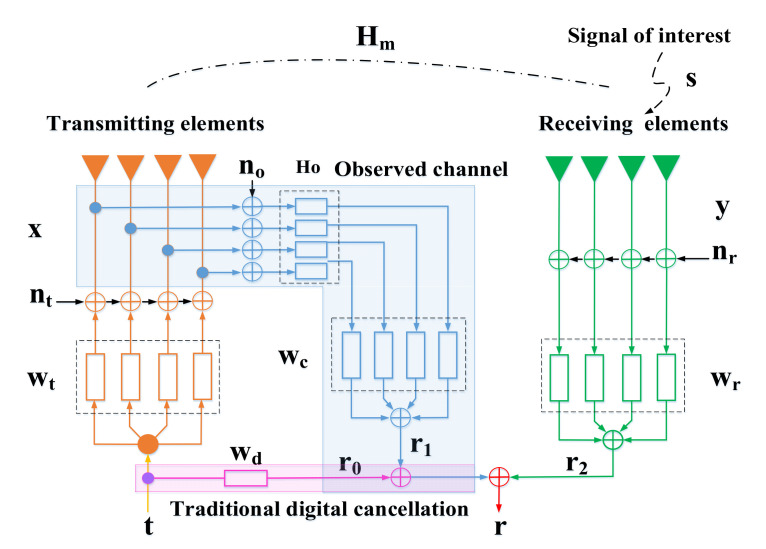
Block diagram of the ALSTAR array cancellation architecture.

The ALSTAR architecture is shown in Figure 1, the symbol $t\in {\mathbb{Q}}^{J\;\times \;1}$ means the transmit signal vector and $r\in {\mathbb{Q}}^{K\;\times \;1}$ denotes the received signal vector. The parameters $w_{t}\in {\mathbb{Q}}^{J\;\times \;1}$, $w_{r}\in {\mathbb{Q}}^{K\;\times \;1}$, and $w_{c}\in {\mathbb{Q}}^{J\;\times \;1}$ are the weights of transmit beamforming, receive beamforming, and adaptive cancellation filters, respectively. $H_{m}\in {\mathbb{Q}}^{K\;\times \;J}$ and $H_{o}\in {\mathbb{Q}}^{J\;\times \;J}$ are the characteristic matrix of the coupled channel and the observation channel, respectively. J and K are the number of transmitting channels and receiving channels, respectively. The signal x at time index n can be written as follows:

(1) $$x(n)\;=\;w_{t}\cdot t(n)\;+\;n_{t}(n)$$

where t(n) is the expected signal to be transmitted and $E\left[|t(n){|}^{2}\right]\;=\;1$. $n_{t}\in {\mathbb{Q}}^{J\;\times \;1}$ is the complex additive white gaussian noise with zero-mean. The signal y is mainly composed of two parts, one is the self-interference signal coupled by the transmit signal through the coupling channel, and the other is the signal of interest s.

(2) $$y(n)\;=\;H_{m}\cdot x(n)\;+\;s(n)$$

The receiving noise $n_{r}\in {\mathbb{Q}}^{K\;\times \;1}$ is mixed with the received signal y. After being processed by the receiving beamformer, they are accepted by the receiver. Therefore, the received signal can be shown as follows:

(3) $$r_{2}(n)\;=\;w_{r}^{H}\cdot (n_{r}(n)\;+\;y(n))$$

In the case of the ALSTAR architecture, the final received signal r(n) after the cancellation can be expressed as follows:

(4) $$r(n)\;=\;r_{2}(n)\;-\;r_{1}(n)$$

where $r_{1}(n)$ is the reference signal after passing through the observation channel, and its expression can be noted as follows:

(5) $$r_{1}(n)\;=\;w_{c}^{H}[H_{o}(x(n)\;+\;n_{o}(n))]$$

Assuming $w_{c}{}^{H}\;=\;w_{r}^{H}\cdot H_{m}\cdot H_{o}^{-1}$ and combining the Equations (4) and (5), we can get

(6) $$r(n)\;=\;w_{r}^{H}[n_{r}(n)\;+\;s(n)\;-\;H_{m}\cdot n_{o}(n)]$$

where r(n) is composed of three parts: receiving noise $n_{r}(n)$, the signal of interest s(n), and observation noise $n_{o}(n)$. The signal $n_{o}\in {\mathbb{Q}}^{J\;\times \;1}$ is additive white gaussian noise, which obeys the normal distribution, i.e.,

(7) $$n_{o}(n)∼N(0,N_{o})$$

where $N_{o}$ is equal to $diag(w_{t}\cdot w_{t}^{H})/\rho_{r}$ and $\rho_{r}$ denotes the receive dynamic range. Apparently, mitigation of the observation noise and emission noise can be achieved by optimizing the transmitting beamforming weight $w_{t}$ and receiving the beamforming weight $w_{r}$.

### 2.2. Metrics and Optimization Problems

This article optimizes the ALSTAR array design from the perspective of selecting the three best parameters, $w_{t}$, $w_{r}$, and $w_{f}$, to improve the overall performance and simplify the design. For the directional system, the isolation between the transmitter and the receiver changes with each position in the three-dimensional space, so EII is used to accurately measure the isolation between the directional systems, which is defined as follows:

(8) EII = EIRP/EIS

where EIRP and EIS describe the performance of transmitters and receivers, respectively, and their expressions are shown:

(9) $$\mathrm{EIRP}\;=\;P_{t}\cdot G_{t\_total}\;=\;P_{t}\cdot \sum_{i\;=\;1}^{J}w_{t_{i}}g_{t_{i}}(\theta ,\varphi )u_{t_{i}}(\theta ,\varphi )$$

(10) $$\mathrm{EIS}\;=\;P_{r}/G_{r_{\_total}}\;=\;(P_{n_{r}}\;+\;P_{n_{o}})/\sum_{i\;=\;1}^{K}w_{r_{i}}g_{r_{i}}(\theta ,\varphi )u_{r_{i}}(\theta ,\varphi )$$

where $u_{t}$ and $u_{r}$ are the manifold vectors of the transmitting and receiving array, respectively, and $g_{t_{i}}$ and $g_{r_{i}}$ are gain of the transmitting and receiving elements, respectively. θ and φ represent the elevation angle and azimuth angle, respectively. The scalars of $P_{n_{r}}$ and $P_{n_{o}}$ are the receiver and observation noise, respectively.

(11) $$P_{n_{r}}\;=\;(H_{m}diag(w_{t}w_{t}^{H})H_{m}^{H})/\rho_{r}$$

(12) $$P_{n_{o}}\;=\;w_{r}^{H}\cdot \{[diag(H_{m}w_{t}w_{t}^{H}H_{m}^{H})]/\rho_{r}\;+\;[diag(H_{m}diag(w_{t}w_{t}^{H})H_{m}^{H})]/\rho_{r}\rho_{t}\;+\;\vartheta^{2}\cdot eye(J)\}\cdot w_{r}$$

where the symbol $\vartheta^{2}$ is the noise floor. From Equations (8)–(10), it can be seen that the EII, EIRP, and EIS of the array are closely related to the transmit beamforming weight $w_{t}$ and receive beamforming weight $w_{r}$. Thus, we optimize the transmit and receive beamformers to obtain the desired EII, EIRP, and EIS. In frequency-modulated continuous-wave radars, it is possible to lower EII to achieve higher EIRP and gain. To achieve this, the weight $w_{f}\;=\;\left[w_{{f\;}_{EII}},w_{{f\;}_{EIRP}},w_{{f\;}_{EIS}}\right]$ is proposed to trade between the EII, EIRP, and EIS in this paper. Therefore, the ALSTAR array can be designed via optimizing three parameters (i.e., $w_{t}$, $w_{r}$, and $w_{f}$) to achieve a higher EII with the premise of relatively high radiation efficiency and better receiving gain. The value ranges of them are as follows:

(13) $$\left\{\begin{array}{l} 0\;\leq |\;w_{t}\;|\leq \;1;\;0\;\leq ∠\;w_{t}\;\leq \;2\pi \\ 0\;\leq |\;w_{r}\;|\leq \;1;\;0\;\leq ∠\;w_{r}\;\leq \;2\pi \\ ||w_{f}|{|}_{1}\;=\;1 \end{array}\right.$$

as the EII, EIRP, and EIS of the ALSTAR array have a theoretical upper bound, their optimization results must be smaller than it. The scope of EII, EIRP, and EIS is given as follows:

(14) $$\left\{\begin{array}{l} \mathrm{EII}\;\leq \;\frac{P_{t}\cdot G_{t\_total}\cdot G_{r\_total}}{\vartheta^{2}}\\ \mathrm{EIRP}\;\leq \;P_{t}\cdot G_{t\_total}\\ \mathrm{EIS}\;\leq \;\frac{\vartheta^{2}}{G_{r\_total}} \end{array}\right.$$

thus, the objective function or fitness function complying with the above conditions can be provided as follows:

(15) $$Fitness\;=\;\min(w_{f}\;\times \;fitness^{T})\;\pm \;\zeta$$

where fitness signifies the total objective function value, which consists of three parts, as shown in Equation (16). The coefficient $w_{f}$ indicates the weight of each part. The interaction of the three goals can be achieved by modifying their weights. As the fault tolerance value, ζ is used to promote the iterative process of the algorithm.

(16) $$\begin{array}{ll} fitness\; & =\;abs(\mathrm{EII}\;-\;\frac{P_{t}\cdot G_{t\_total}\cdot G_{r\_total}}{\vartheta^{2}})\\ & +\;abs(\mathrm{EIRP}\;-\;P_{t}\cdot G_{t\_total})\\ & +\;abs(\mathrm{EIS}\;-\;\frac{\vartheta^{2}}{G_{r\_total}}) \end{array}$$

## 3. Our Proposed Algorithm

In response to the problems of traditional BSO algorithms, we consider amending the BSO from the presetting initial value, grouping strategy, individual creation, and individual update to enhance the convergence speed, global search capability, and reduce the operating overhead. We call the improved algorithm the adaptive random grouping quantum brainstorming optimization (ARGQBSO), which is a hybrid algorithm that combines quantum computing and classical BSO. Its performance-improvement is mainly reflected in four aspects.

### 3.1. Preset Initial Value

The initial value of the transmitting and receiving beamformer is directly related to the convergence speed of the algorithm. We reduce the search space of the algorithm without reducing the performance by setting an appropriate initial value. In the original BSO algorithm, the initial range of each idea (individual) is a random number of [0, 1]. According to the principle of maximum entropy, the maximum EIRP value can be obtained when the $w_{t}$ is uniformly distributed. When $w_{t}\;=\;0$, the vintage EIS can be obtained. In order to find a result that meets the actual needs among EII, EIRP, and EII, the optimal solution range of $w_{t}$ must appear in $\left(0,\frac{1}{\hbar }\right)$. ħ is the number of transmitting elements. Obviously, by setting the initial value in this way, the search space has been reduced $1\;-\;\frac{1}{\hbar }$. In addition, the initial value of the transmit and receive beamforming weights should be related to the number of array elements and the array manifold vector. Its expression is as follows:

(17) $$Population\;=\;[\frac{1}{\hbar }u_{t};\frac{1}{ƛ}u_{r}]*X_{\min}\;+\;(X_{\max}\;-\;X_{\min}).*rand(D,N)$$

where the symbol ħ and ƛ represent the number of transmitting and receiving elements. $X_{max}$ and $X_{\min}$ imply the upper boundary and lower boundary of the population, respectively, D implies the dimension of the population, and N denotes the size of the population.

### 3.2. Random Grouping

**Figure 2:**
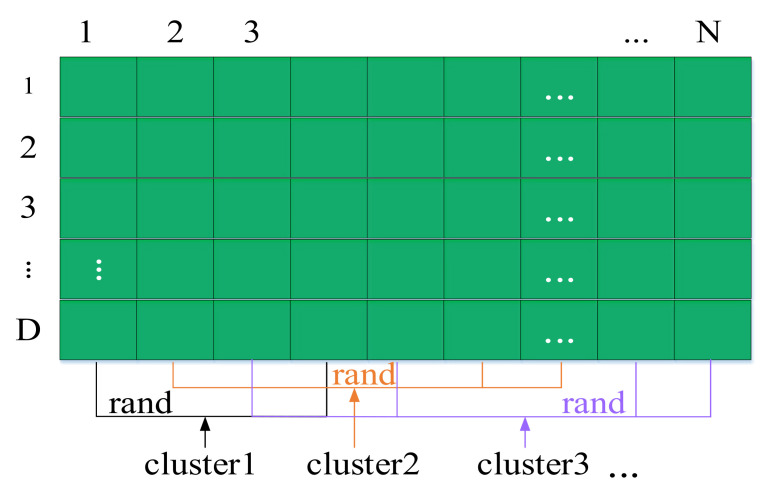
Schematic diagram of random grouping.

Random grouping is used to replace the original k-means, which avoids calculating the distance between different individuals and reduces the calculation. The population random grouping process is shown in Figure 2.

D depends on the number of elements. The cluster represents the same kind of individual population. The specific process is as follows: $\ell_{1}$ individuals are randomly selected from populations N, and recorded as cluster1. Similarly, randomly select $\ell_{2}$ individuals from the remaining $\left(N\;-\;\ell_{1}\right)$ individual species and record them as cluster2, and so on. It should be noted that there ℓ is a $1\;\times \;n_{c}$ vector, and ${\left\|\ell \right\|}_{1}\;=\;n_{c}$,$n_{c}$ represents the number of clusters.

### 3.3. Dynamic Probability Function

In the original BSO, there are four ways of individual creation [20], two of which create individuals based on a single cluster center, and the others create individuals based on two cluster centers. The individuals created by the former surround their clusters and have strong local search characteristics, while the individuals created by the latter may appear in the entire space and means the global search. The dynamic probability function $p_{0}$ improves the local and global search performance of ARGQBSO, making the algorithm focus on the global search in the early stage and the local search in the later stage to enhance the convergence speed. The dynamic probability function $p_{3}$ is used in the later stage of the algorithm to avoid missing the global best solution. Their expressions are as follows:

(18) $$\left\{\begin{array}{l} p_{0,t\;+\;1}\;=\;\exp(-\gamma {\left(t/t_{\max}\right)}^{k})\;\times \;p_{0,t}\\ p_{3,t\;+\;1}\;=\;\exp(\gamma {\left(t/t_{\max}\right)}^{k})\;\times \;p_{3,t} \end{array}\right.$$

The meaning of t is the current number of iterations; $t_{\max}$ is the maximum number of iterations; $p_{0,t}$, $p_{3,t}$, and $p_{0,t\;+\;1}$, $p_{3,t\;+\;1}$ are the probabilities of the present and next generations, respectively. γ and κ are positive integers.

### 3.4. Quantum Update

The quantum behavior mechanism is introduced into all individuals so that every individual is transformed from the original classical state to the quantum state. At this time, individuals in the quantum state pass through the quantum revolving gate to update iteratively.

**Figure 3:**
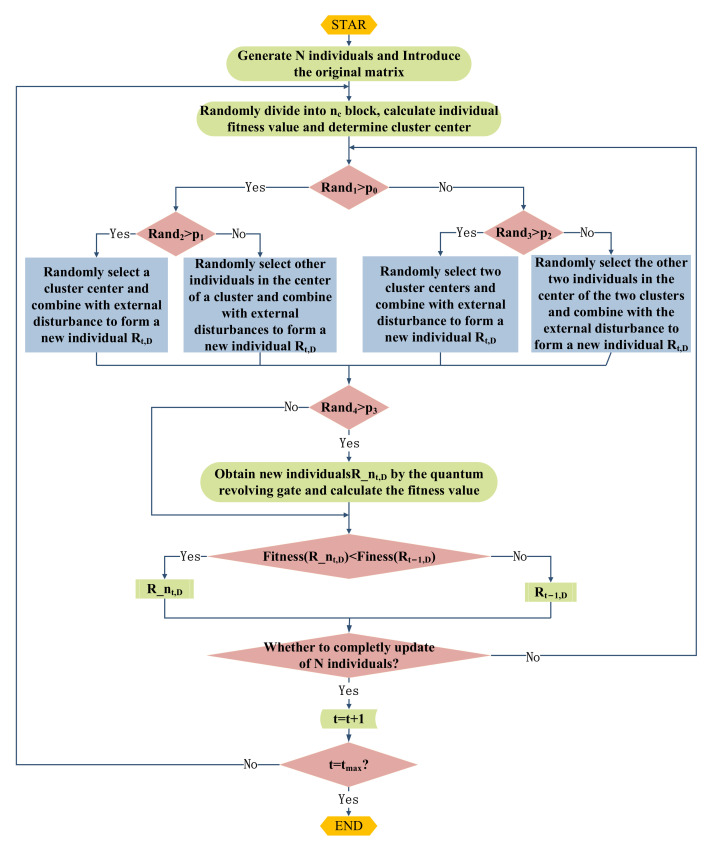
The flowchart of the ARGQBSO algorithm.

Different from [16], in this paper, the individual’s quantum behavior only takes effect in the later stage of the algorithm, and the quantum state is transformed through a dynamic quantum spin gate. As the value of the dynamic probability function is already quite small in the later stage of the algorithm, the individuals created at this time are distributed almost in the center of a cluster, and diversity is completely lost. This is highly unfavorable for solving infinite domain and multi-extreme problems like ALSTAR. In particular, the noise, channel, and transmit power may change with the environment, if the ALSTAR system is running. To automatically adapt to these accidents, a dynamic quantum revolving gate is introduced in the later stage of the algorithm to enhance the global convergence ability in the later stage to avoid missing the best solution. The individuals in the quantum state follow the Equation (19) to update.

(19) $$\begin{array}{l} R\_n_{t\;+\;1,D}\;=\;R_{t,D}\;+\;sign(R_{t,D}\;-\;globe_{t,D})\;\times \;\Delta \Theta \\ \Delta \Theta \;=\;\exp(-\varepsilon \;\times \;\frac{I_{t}}{I_{\max}})\;\times \;\Theta_{0} \end{array}$$

where $R_{t,D}$ and $R_{t\;+\;1,D}$ represent contemporary and next-generation individuals, respectively; $globe_{t,D}$ is the current global optimal individual; ΔΘ is the dynamic revolving gate; and $\Theta_{0}$ represents the initial rotation angle. The flowchart of the ARGQBSO algorithm is shown in Figure 3. The scalars $p_{1}$ and $p_{2}$ are random numbers of [0, 1]. The variables $p_{0}$ and $p_{3}$ are dynamic probability functions, respectively.

## 4. Simulation Results

The performance of the ALSTAR array is measured by the three objectives of EII, EIRP, and EIS. We minimize the fitness function to obtain the best transmit beamforming weight $w_{t}$ and receive beamforming weigh $w_{r}$. The optimization model is given in the second part. As the characteristics of the coupling matrix and antenna gain are directly linked to EII, EIRP, and EIS of the ALSTAR from Equations (8)–(12), it is necessary to design a phased array with high isolation and high gain. Subsequently, based on the coupling matrix of the designed phased array and the pattern data of each element, the effects of the preset initial value, random grouping, dynamic probability function, and quantum update on the proposed algorithm are analyzed in detail. In addition, by comparing the performance of six commonly used optimization algorithms on the ALSTAR array design, the competitiveness of the ARGQBSO algorithm is verified. Finally, we explore the EII, EIRP, and EIS under extreme differences $w_{f}$ using ARGQBSO.

### 4.1. Phased Array with High Isolation

**Figure 4:**
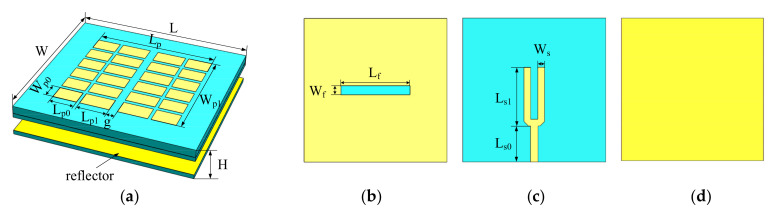
Broadband antenna model: (**a**) 3D view; (**b**) gap structure; (**c**) feed-network; (**d**) metal reflector.

The array element adopts the microstrip antenna fed by slot coupling, and its structures are shown in Figure 4. The microstrip patch is divided into several small pieces in the horizontal and vertical directions to form the series capacitor periodic array loaded with metamaterial patch elements [21]. In addition, loading a reflector on the bottom of the antenna enhances the directivity and front-to-back ratio, without deteriorating the matching performance. The proposed antenna is simulated in ANSYS HFSS, and its optimized parameters are $L\;=\;34.2\;\mathrm{mm};L_{p}\;=\;22.5\;\mathrm{mm};L_{p0}\;=\;5.8\;\mathrm{mm};L_{p1}\;=\;4.6\;\mathrm{mm};L_{f}\;=\;16.8\;\mathrm{mm}\;L_{s0}\;=\;8.8\;\mathrm{mm};L_{s1}\;=\;14\;\mathrm{mm};W\;=\;34.2\;\mathrm{mm};W_{p0}\;=\;3.2\;\mathrm{mm};W_{p1}\;=\;23.2\;\mathrm{mm};W_{f}\;=\;2.1\;\mathrm{mm};W_{s}\;=\;1.6\;\mathrm{mm};H\;=\;5.235\;\mathrm{mm};g\;=\;0.83\;\mathrm{mm}.$

**Figure 5:**
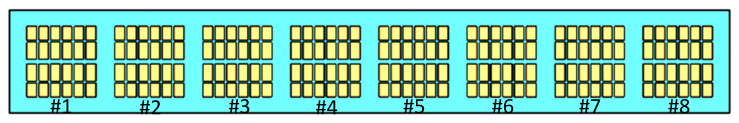
Schematic diagram of the broadband digital phased array structure.

**Figure 6:**
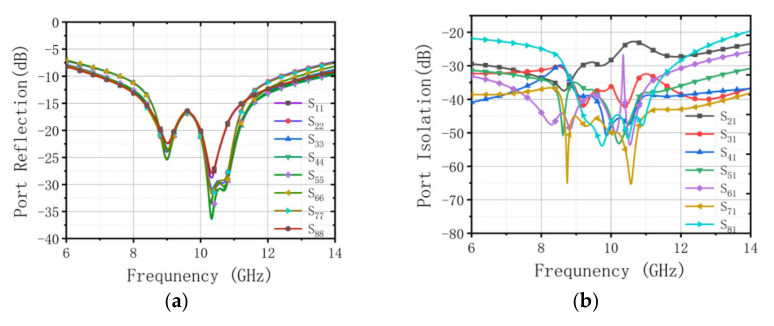

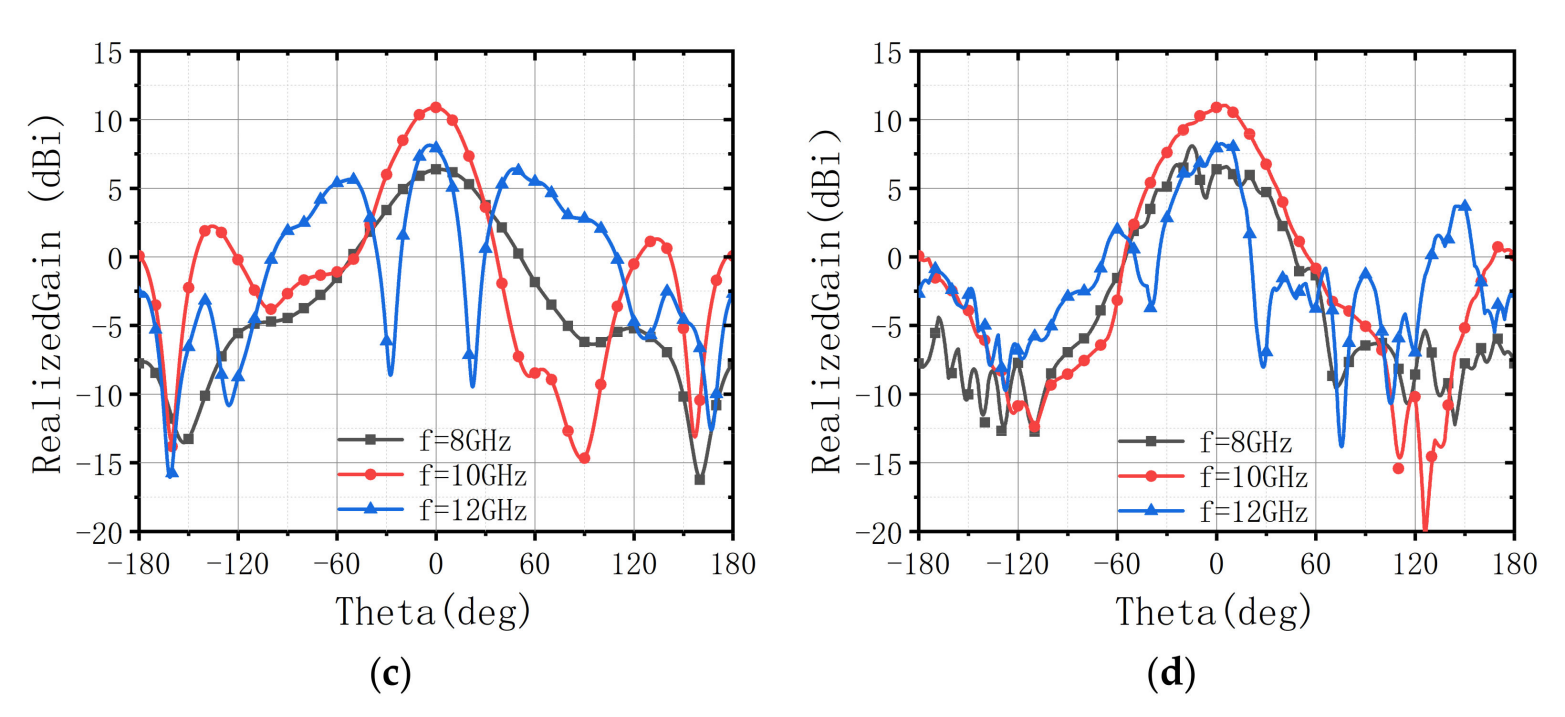
(**a**) The port reflection parameters of the array. (**b**) Port isolation parameters of the array. (**c**) E-plane pattern of the first element. (**d**) H-plane pattern of the first element.

The STAR array is shown in Figure 5. It is composed of eight designed broadband antenna elements evenly arranged at a pitch of 0.55 $\lambda_{0}$. $\lambda_{0}$ is the wavelength in free space. The simulation results of the phased array are shown in Figure 6. Part of the simulation data is shown in Appendix A. The impedance bandwidth of this antenna is 6.92~13.45 GHz under the condition that the port reflection coefficient is less than −10 dB, and the isolation between the adjacent antennas is about 25 dB in the whole X-band.

In the ALSTAR array, we assume that the 1~4 elements on the left of the phased array are transmitting antennas and the 5~8 are receiving antennas. The coupling matrix $H_{m}$ is used to describe the state of electromagnetic waves from the transmitter to the receiver and it is a K × J matrix. K and J represent the number of receiving and transmitting antennas, respectively. $H_{m}$ is written as follows:

(20) $$H_{m}\;=\;\left[\begin{array}{cccc} S_{51} & S_{52} & S_{53} & S_{54}\\ S_{61} & S_{62} & S_{63} & S_{64}\\ S_{71} & S_{72} & S_{73} & S_{74}\\ S_{81} & S_{82} & S_{83} & S_{84} \end{array}\right]$$

In practice, adaptive beamforming and digital cancellation requires a great estimate of the mutual coupling channel in the ALSTAR array in order to consider the coupling caused by the time-varying environment or external interference. Yet it can be considered that the coupling matrix varies slowly in most scenarios. For example, there are not many fast-varying scenes in communications. In the radar, we mainly focus on specific targets, and may not be scene varies. In this case, the slow vary of the coupling matrix can be ignored for the time being.

### 4.2. Algorithm Performance Analysis

#### 4.2.1. Analysis of the Role of Improved Operations

In order to expose the effects of preset initial values, random grouping, dynamic probability functions, and quantum updates on the AGRQBSO algorithm, we separately analyzed the benefits of each operation. Before that, we incorporated the algorithm into the metrics and optimization model of the ALSTAR array established in Section 2.2. The ALSTAR antenna adopts the phased array designed above, and the system’s dynamic range of transmitting and receiving channels is $\rho_{t}\;=\;40$ dB and $\rho_{r}\;=\;80$ dB, respectively. The noise floor of the receiving channel is −81 dB, which is obtained by the 1000 MHz bandwidth channel with a 3 dB noise figure. The objective function and the boundary range of the parameters are given in Section 2. The experiments were executed in MATLAB software (Version: R2021a) and all comparative experiments were executed on a desktop PC with an Intel Core i7-8700 CPU processor @ 3.20 GHz, 16GB RAM, under the Windows10 64-bit OS.

**Figure 7:**
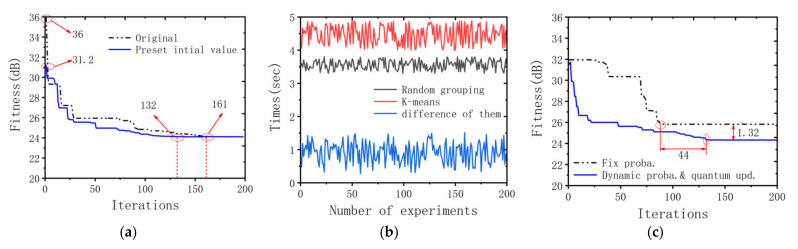
(**a**) Comparison of the fitness value with/without preset initial value. (**b**) The influence curve of grouping method on algorithm running time. (**c**) Comparison of the fitness value of fixed probability and dynamic probability density function and quantum update.

We first analyzed the contribution of the preset initial value operation to the ARGQBSO algorithm. As mentioned earlier, the preset initial value reduces the search space of the algorithm and can speed up the convergence of the algorithm. Figure 7a shows the average result of the convergence curve with or without preset initial value operation by repeating the experiment 200 times. It can be seen that since the search space is compressed $1\;-\;\frac{1}{\hbar }$, the algorithm using preset initial values only takes 132 iterations to the state of convergence. Compared with the original scheme, the number of iterations is reduced by 29. In addition, the initial fitness value of the algorithm using the preset initial value is 4.8 dB smaller than the original algorithm. This hints that the preset the initial value places the algorithm in a better position in the actual stage and speeds up the convergence.

The contribution of the random grouping to the ARGQBSO algorithm is mainly to reduce the complexity of the clustering. In order to intuitively feel the reduction of the algorithm complexity by the random grouping, Figure 7b shows the running time of the algorithm using random grouping and K-means grouping in 200 experiments. The result of the experiment implies that the time of the random grouping algorithm is reduced in the range of 0.3145 to 1.4852 s.

The usefulness of the dynamic probability function to the algorithm is mainly reflected in the adjustment of the individual generation mode. By controlling the proportion of individuals participating in the global and local search, the global search is strengthened in the early stage of the algorithm to find a better position, and the local search is strengthened in the later stage of the algorithm to speed up the convergence speed.

The essence of the quantum update mechanism is to alter the evolution step length of the newly generated individual, but it is different from the individual update step length of the original BSO. It is a dynamic quantum rotation update, and the generated individuals surround the current global optimum. The contribution of dynamic probability density function and quantum update to the algorithm is reflected in the accuracy of the solution. Figure 7c shows the average results of 200 experiments. The algorithm using dynamic probability density function and quantum update is 1.32 dB smaller than the fitness value of the fixed probability, indicating that the accuracy of the former is better than the latter.

On the other hand, the number of iterations that the fixed probability algorithm uses to reach the convergence state is 44 less than that of the dynamic probability function and quantum update algorithm. This shows that the fixed probability algorithm has an insufficient global convergence ability and is easy to fall into the local optimal region.

#### 4.2.2. Comparison of ARGQBSO with Other Algorithms

We explore the comprehensive performance of ARGQBSO, BSO, PSO, AO, GA, and DE in terms of the objective function value, convergence speed, and running time. What is different from [8] is that the $w_{t}$ and $w_{r}$ obtained by the ARGQBSO can be applied to all elevation angles, while there is no need for a set of $w_{t}$ and $w_{r}$ at every elevation angle. The advantage of this scheme is to reduce the processing time of beamforming and the calculation cost of the DSP chip.

**Figure 8:**
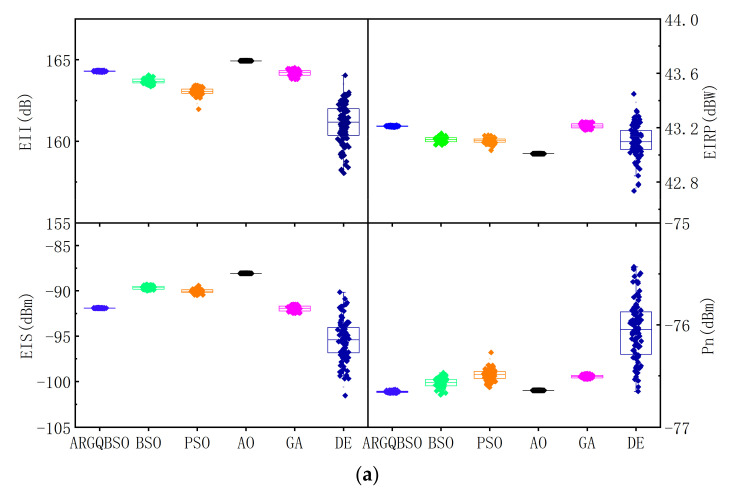

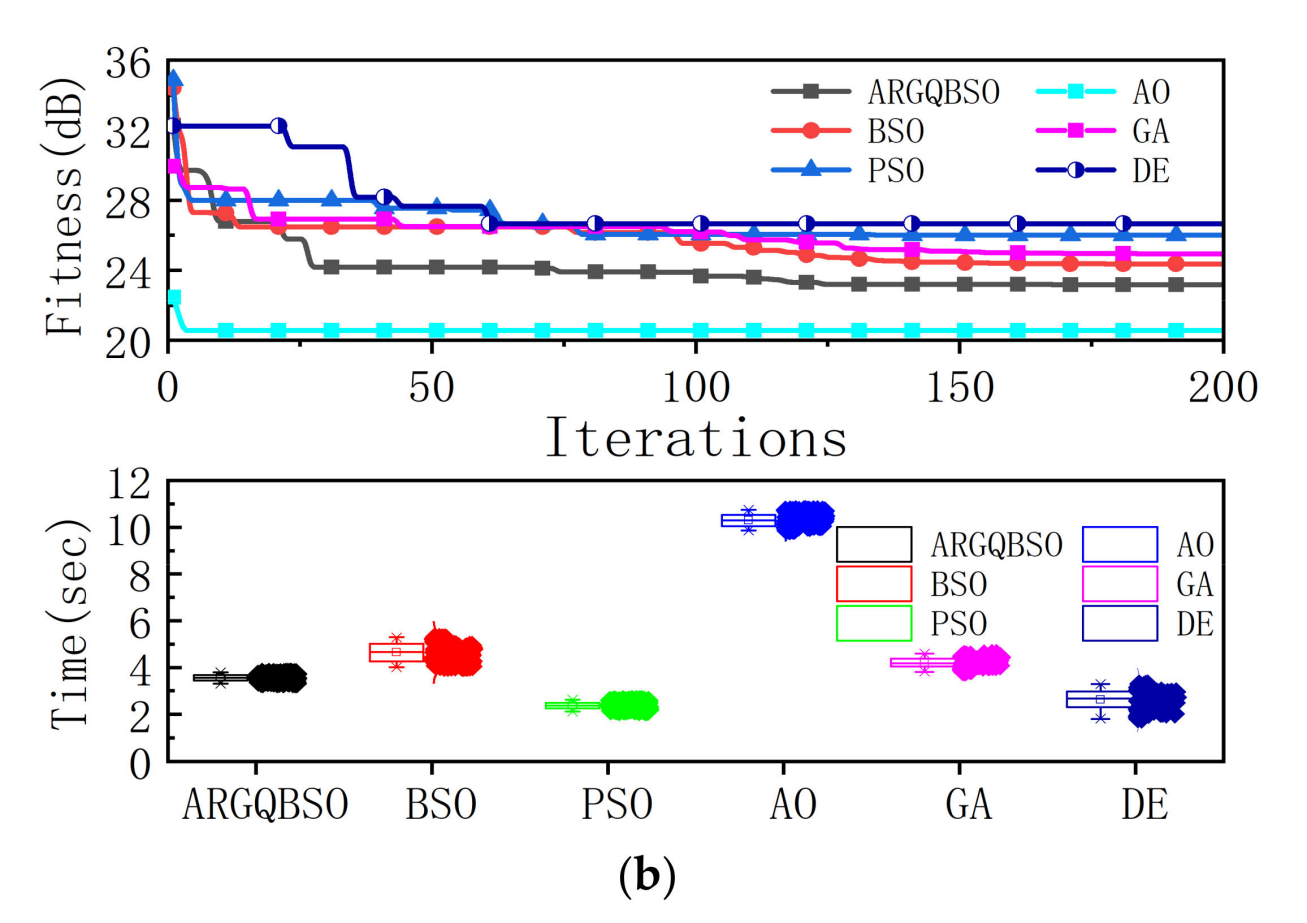
(**a**) Box plot of the three algorithms for EII, EIRP, EIS, and noise floor $P_{n}$. (**b**) Iterative curve and operation time of the four algorithms.

The parameter settings of the six algorithms is shown in Table 1. Figure 8a clearly shows the distribution of EII, EIRP, EIS, and noise floor $P_{n}$ in 100 times independent runs with $P_{t}\;=\;1000$ W, and the convergence process and running time of the six algorithms are shown in Figure 8b. On the one hand, compared with the classic BSO and PSO, ARGQBSO has advantages in robustness, global optimization capability, and convergence speed. Although GA has great global optimization capabilities and solution accuracy, it does not have the advantage in terms of robustness and complexity. DE can occasionally achieve great solution accuracy, but its robustness is most poor. On the other hand, the AO can find a better solution (smaller fitness value) because of the EII function satisfying the generalized Rayleigh entropy. However, AO must construct EII equations in two directions for alternating iterations, which nearly doubles the number of calculations relative to the ARGQBSO algorithm, and its running time and calculation amount are the most complicated. This may be the reason for limiting the application of AO in engineering. Therefore, compared to other synthesis approaches, ARGQBSO is claimed as a better trade-off in terms of stability, solution time, and solution accuracy. In addition, the structure of ARGQBSO allows for many functions to be extended, for example, it can optimize the directivity of the EII pattern, the beam width, and trade EII, EIRP, and EIS among them. Thus, the proposed algorithm demonstrates its distinctive competitive advantages in terms of complexity, accuracy, and reliability.

### 4.3. Design of ALSTAR Array by ARGQBSO

In order to verify the advantages of the solution in this article, we employ the proposed ARGQBSO algorithm to design the ALSTAR array, and explore the range of values of EII, EIRP, and EIS under different $w_{f}$. In ARGQBSO, every individual in the population is mapped to the transmitting and receiving beamforming weights as a feasible solution. The mapping relationship between the feasible solution and the transmitting and receiving beamforming weights are as follows, where globe is the global optimal solution with 2 × (J + K) dimensions, which contains the weights of transmitting and receiving beamforming.

(21) $$\begin{array}{l} w_{r}\;=\;u_{r}\;*\;(globe(1:J)\;+\;j\;*\;globe(J\;+\;1,2J))\\ w_{t}\;=\;u_{t}\;*\;(globe(2J\;+\;1:2J\;+\;K)\;+\;j\;*\;globe(2J\;+\;K\;+\;1,2(J\;+\;K))) \end{array}$$

**Figure 9:**
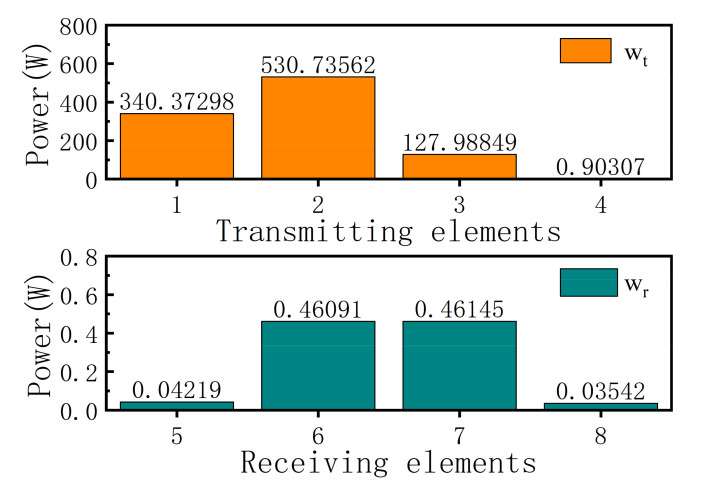
Transmit and receive beamforming vector.

Thus, the transmit and receive beamforming weights can be extracted by the formula (21). We set the total transmitting power of the ALSTAR array as 1000 W and the weight $w_{f}$ as $\left[0.6,0.15,0.25\right]$. The optimization results of the transmitting beamforming weight $w_{t}$ and receiving beamforming weight $w_{r}$ by the ARGQBSO are shown in Figure 9. Here, the signal power transmitted by the four transmit channels is 1, 2, 3, and 4, respectively. The powers of the four receiving channels are 5, 6, 7, and 8, respectively. By using the obtained transmit beamforming and receive beamforming weights, the EII, EIRP, and EIS of the array can be calculated.

**Figure 10:**
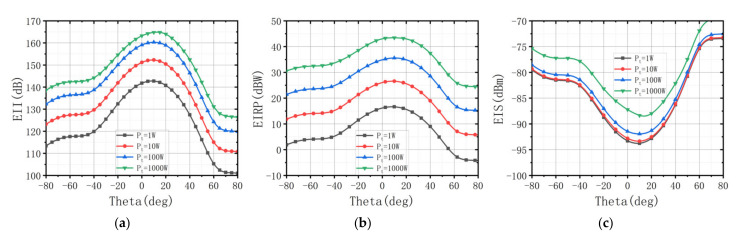
(**a**) EII with different transmit power. (**b**) EIRP with different transmit power. (**c**) EIS with different transmit power.

Figure 10 shows EII, EIRP, and EIS at 10 GHz with $P_{t}$ from 1 W to 1000 W at $w_{f}\;=\;[0.6,0.15,0.25]$. In the case of a transmit power of 1000 W, EII reaches 164.9 dB, EIRP is 44.2 dBm, and EIS is −87.3 dBm. This result verifies that the proposed ARGQBSO algorithm can design a great ALSTAR array. From another perspective, it can be clearly understood that the EII and EIRP of the ALSTAR system gradually grow as the transmit power increases, while the EIS deteriorates. Moreover, accompanied by the increase in transmit power, the step size of the EII decreases, and EII tends to the theoretical boundary.

**Figure 11:**
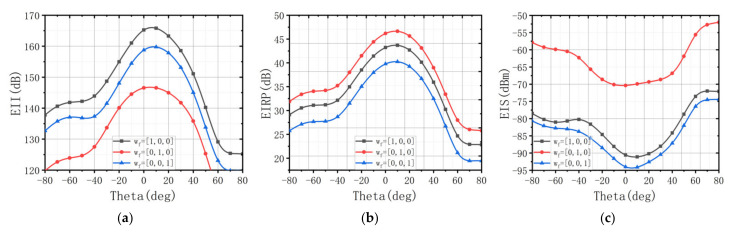
(**a**) EII with different $w_{f}$. (**b**) EIRP with different $w_{f}$. (**c**) EIS with different $w_{f}$.

In FMCW radar, EIS is not allowed to exceed a certain threshold, otherwise, the receiver will not be able to operate properly. We may enhance EIS by lowering EII and EIRP on the weight of $w_{f}$. Figure 11 shows EII, EIRP, and EIS of extreme weight conditions in 10 GHz with 1000 W of transmit power. $w_{f}\;=\;\left[1,0,0\right]$ indicates that there is only EII in the objective function, $w_{f}\;=\;\left[0,1,0\right]$ means that there is only EIRP in the objective function, and $w_{f}\;=\;\left[0,0,1\right]$ means that there is only EIS in the objective function. By setting three extreme $w_{f}$ situations, it can be seen clearly that the EII fluctuates between 146.7~166.8 dB, EIRP fluctuates from 40.2 to 47.1 dBW, and EIS fluctuates from −70.3 to −94.6 dBm. This considerable adjustment range makes it possible for ALSTAR to provide multi-scenario applications. On this basis, we can design an ALSTAR array to realize the trade of EII, EIRP, and EIS by changing the parameter $w_{f}$ for different scenarios. This is to prevent engineers from considering only the isolation, while ignoring the performance of the transmitter and receiver itself when they are designing the STAR array.

## 5. Conclusions and Future Work

A robust design for the ALSTAR array was proposed in this paper. The transmit and receive beamforming weights obtained by the ARGQBSO algorithm are independent of the scanning angles, which reduces the computational complexity and maintains the excellent overall performance of the ALSTAR array. It expands the scope of application of transmitting and receiving beamforming weights and has a high robustness. ARGQBSO is an improved version of the original BSO algorithm. Its improvements include four aspects: initial value, random grouping mechanism, dynamic probability function, and quantum computing. Experimental results show that the preset initial value shortens the search range and speeds up the convergence Random grouping reduces the complexity of the algorithm. Dynamic probability function and quantum update improve the accuracy of the algorithm. In addition, the solution time, accuracy, and robustness of the proposed algorithm are claimed as a better trade-off, compared to other synthesis approaches. The simulated results based on an eight-element phased array indicate that the array achieves 166.8 dB of peak EII, 47.1 dBW of peak EIRP, and −94.6 dBm of peak EIS at $P_{t}\;=\;1000\;W$ with $w_{f}\;=\;[1,0,0],w_{f}\;=\;[0,1,0],w_{f}\;=\;[0,0,1]$, respectively. In addition, EII fluctuates between 146.7~166.8 dB, EIRP fluctuates between 40.2~47.1 dBW, and EIS fluctuates between −70.3~−94.6 dBm. The results verify that the ARGQBSO is competitive in the ALSTAR application, and it trades among EII, EIRP, and EIS to satisfy the needs of different scenarios. The ARGQBSO algorithm provides engineers with a concise way to design massive transmit and receive arrays, and to achieve a superior overall performance of the ALSTAR array.

In the future, ARGQBSO will be used to solve more optimization problems in engineering design and other fields. Furthermore, some other strategies will be developed to further optimize the performance of the proposed algorithm.

## Figures and Tables

**Table 1:** The parameter settings of four algorithms.

Algorithm Types	Parameter Setting	Reference
BSO	$p_{r00}\;=\;0.2;$ $\;p_{r0}\;=\;0.8;$ $\;p_{r01}\;=\;0.4$	[15]
	$p_{r02}\;=\;0.5;$ M = 10; N = 60	
PSO	w: 0.9–0.4.4; $\;c_{1}\;=\;c_{2}\;=\;2;$ N = 60;	[22]
AO	error accuracy = 0.001; Itern = 200	[11]
GA	$p_{c}\;=\;1;$ $\;p_{m}\;=\;0.05;$ N = 60	[23]
DE	F = 0.5; CR = 0.5; N = 60	[24]
Proposed	$\begin{array}{lll} p_{0}:\;0.9–0.4; & \;p_{3}:\;0.4–0.8;\\ & p_{1}\;=\;0.7;\\ p_{2}\;=\;0.5; & M\;=\;10; & N\;=\;60 \end{array}$	This work

**Figure A1:**
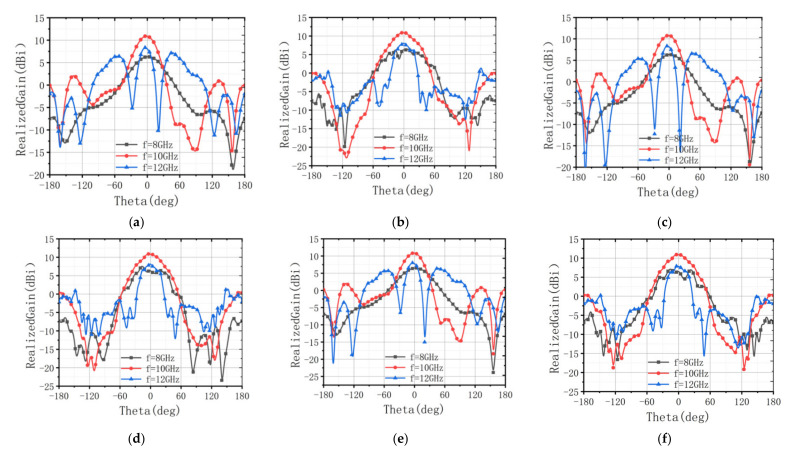
(**a**) E-Pattern of the second element. (**b**) H-Pattern of the second element. (**c**) E-Pattern of the third element. (**d**) H-Pattern of the third element. (**e**) E-Pattern of the fourth element. (**f**) H-Pattern of the fourth element.

## Data Availability

Not applicable.

## Appendix A

The pattern of array elements 2–4 is shown in Additional Figure A1. Due to the high isolation performance of the designed phased array, the pattern characteristics of each array element are almost similar, which is conducive to beam forming. In addition, as the array elements numbered 5–8 are symmetrical to the center of array elements 1–4, their pattern data are almost the same, and they are not listed here.
